# Thermal Stress Induces Metabolic Responses in Juvenile Qingtian Paddy Field Carp *Cyprinus carpio var qingtianensis*

**DOI:** 10.3390/ani12233395

**Published:** 2022-12-02

**Authors:** Yuhan Jiang, Xiangbing Cheng, Junjie Lu, Guanhong Xu, Qigen Liu, Jiamin Sun

**Affiliations:** 1 Centre for Research on Environmental Ecology and Fish Nutrition of the Ministry of Agriculture and Rural Affairs, Shanghai Ocean University, Shanghai 200120, China; 2 Key Laboratory of Exploration and Utilization of Aquatic Genetic Resources, Ministry of Education, Shanghai Ocean University, Shanghai 200120, China; 3 Shanghai Engineering Research Center of Aquaculture, Shanghai Ocean University, Shanghai 200120, China; 4 Key Laboratory of Integrated Rice-Fish Farming, Ministry of Agriculture and Rural Affairs, Shanghai Ocean University, Shanghai 200120, China; 5 Yugong Ecological Agriculture Technology Co., Ltd., Lishui 323000, China

**Keywords:** carp, paddy field, metabolomics, liver, thermal stress

## Abstract

**Simple Summary:**

For many aquatic animals, temperature is an important environmental factor that influences how they develop, reproduce, behave, and survive. For both economic and ecological reasons, the Qingtian paddy field carp (*Cyprinus carpio var qingtianensis*) is an important species of fish in China. It is an important aquaculture fish suitable for rice–fish coculture systems. In the current study, we utilized metabolomics to investigate the impact of differential metabolites and metabolic pathways in Qingtian paddy field carp liver tissue under thermal stress. Our findings clarify the molecular regulatory mechanism of PF carp adaptation to thermal stress at the metabolic level and offer novel hypotheses for the biological regulation of many other fish species that generate thermal stress.

**Abstract:**

Extreme fluctuations in water temperature lead to significant economic losses for the aquaculture industry. *Cyprinus carpio var qingtianensis* (locally called Qingtian paddy field carp), is a local variety commonly found in Zhejiang province, China. Unlike traditional aquaculture environments, the water temperature range between day and night in the rice field environment is much larger, and the high temperature in summer may exceed the growth threshold of fish because there is no manual intervention; therefore, the study of how the Qingtian paddy field carp (PF carp) adapts to high-temperature conditions can shed light how the species adapt to the rice field environment. To investigate the molecular mechanisms of this fish under thermal stress, the liver metabolomics of Qiangtian paddy field carp (PF carp) were analyzed. In this study, metabolomics was used to examine the metabolic reaction of PF carp (102 days old, 104.69 ± 3.08 g in weight, 14.65 ± 0.46 cm in length) at water temperatures of 28 °C (control group, CG), 34 °C (experimental group (EG) 34), and 38 °C (EG38). The results show that 175 expression profile metabolites (DEMs), including 115 upregulated and 60 downregulated metabolites, were found in the CG vs. EG34. A total of 354 DEMs were inspected in CG vs. EG38, with 85 metabolites downregulated and 269 metabolites upregulated. According to the pathway enrichment study, various pathways were altered by thermal stress, including those of lipid, amino-acid, and carbohydrate metabolism. Our study presents a potential metabolic profile for PF carp under thermal stress. It also demonstrates how the host responds to thermal stress on a metabolic and molecular level.

## 1. Introduction

Temperature is a crucial ecological component that affects fish life [1]. Various studies have shown that fluctuations in water temperature impact fish development, sexual reproduction, mobility, behavior, and metabolism [2,3,4,5,6]. Typically, fish can adjust to the encompassing temperature around them and keep up homeostasis by rebalancing their behavioral, physiological, biochemical, and molecular processes [7]. However, fish health may suffer greatly at extreme water temperatures. On the other hand, a rise in temperature may promote their growth [8,9]. Thermal stress is produced and the adaptive ability is even disrupted when the temperature rises above the appropriate growth temperature, which results in an imbalance in the body metabolism [10,11,12]. Fish are more prone to endure high-temperature stress in aquaculture, especially in paddy habitats where high temperatures are frequently observed, especially during the summer, changing their internal homeostasis, ecology, and behavior [13]. Several studies have discovered alterations in serum metabolic signals and the activity of enzymes, as well as gene expression in fish exposed to high temperatures. The common carp’s (*Cyprinus carpio*) swimming ability was also significantly affected by temperature [14,15]. Studies on fish exposed to high temperatures have focused on the expression of stress proteins, e.g., the heat-shock protein family and lactate dehydrogenase genes [16,17,18]. In addition, several researchers have revealed the effects of thermal stress on Nile tilapia (*Oreochromis niloticus*), turbot (*Scophthalmus maximus*), grass carp (*Ctenopharyngodon idellus*), Manchurian trout (*Brachymystax lenok*), and black rockfish (*Sebastes schlegelii*) adaptation mechanisms through transcriptomics, metabolomics, and other molecular biology techniques [19,20,21,22,23].

The Qingtian rice–fish coculture system was designated as one of the first pilot “Globally Important Agricultural Heritage Systems (GIAHS)” in 2005 by the Food and Agriculture Organization (FAO) of the United Nations. The rice–fish coculture system has been passed down over a long history of over 1200 years [24]. One variety of carp, known as the Qingtian paddy field carp (*Cyprinus carpio var qingtianensis*), is particularly well suited to the system [25]. Most research on PF carp has been performed on topics such as the skin color of the fish, genetic diversity, the connection between rice and fish, and how the fish adjusts to low dissolved oxygen levels [26,27,28]. When compared with traditional aquaculture, the water environment in rice fields is quite different. The relatively shallow water, the substantial fluctuations in day time and night time temperatures (37.9 ± 1 °C and 22.2 ± 0.2 °C, respectively), and the variances in the anoxic range (DO 4 mg/L) are the most striking distinctions. These differences are especially noticeable in the summer. The development of high heat tolerance in PF carps may occur as a consequence of the long-term coculture of rice and fish. However, the mechanism of its adaptability to high temperatures remains unknown.

The liver is one of the functional organs in fish, and it is responsible for a significant part of metabolic homeostasis [29]. It is the primary location where carbohydrates, proteins, and lipids are synthesized, metabolized, stored, and redistributed throughout the body [30]. Although the liver is physically insulated from environmental variables, its reactions may still be apparent because of its crucial function in metabolism and energy storage [31]. Therefore, the liver is typically used as a biological target in studies pertaining to the monitoring of the environment and studies pertaining to the stress response. Previous research studied differences in seasonal reproductive strategies and energy storage patterns among different species of freshwater fish using livers from seven species of freshwater and estuarine small fish from Atlantic Canada [32]. Recently, substantial research has been performed on how heat stress affects fish livers [33,34,35,36]. However, less attention has been paid to metabolic changes in the PF carp liver caused by thermal stress.

**Figure 1:**
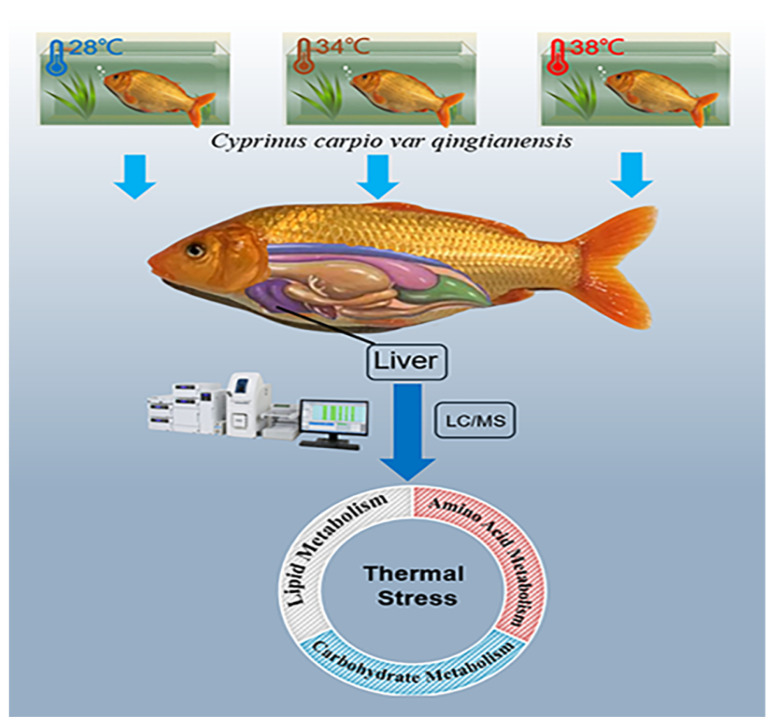
The experimental model of the effects of thermal stress on liver metabolomics of *Cyprinus car pio var qingtianensis*.

Metabolomics is the large-scale study of metabolites or tiny compounds within cells, biofluids, tissues, and organisms [37]. Metabolome analysis can implement qualitative and quantitative analyses of all low-molecular-weight metabolites shown in biological samples, in addition to checking for metabolites that demonstrate substantial biological differences between groups [38]. Variations in metabolites can reflect the operation of upstream regulatory genes; therefore, in addition to reflecting molecular mechanisms, it can be used to analyze potential biological effects [10]. It has been used effectively to study disease transmission and fish toxicity [39,40,41]. Furthermore, many studies have shown that thermal stress affects the metabolism of aquatic animals [6,10,23]. In one case, Yang et al. (2020) investigated the effect of thermal stress on the metabolic level of turbot (*Scophthalmus maximus*). The results showed that heat stress reduced the levels of glycosylated amino acids in turbot, and the differential metabolites were mainly enriched in carbohydrate and amino-acid metabolism [42]. In order to investigate the effects of heat stress on PF carp metabolism, in this study, varying temperatures were set in the experimental group (34 °C and 38 °C) and the control group (28 °C) according to the results of a preliminary experiment. We hypothesized that thermal stress may affect PF carp metabolites and metabolic pathways; therefore, we applied liquid chromatography/mass spectrometry (LC–MS) to explore the changes in metabolites in juvenile PF carp liver tissue after exposure to high temperatures and to investigate metabolic pathways. Our findings provide insight into the molecular mechanisms that regulate metabolism in PF carp under high-temperature stress, as well as provide new insight into fish adaptation to the environment in the rice–fish coculture system (Figure 1).

## 2. Materials and Methods

### 2.1. Fish Preparation and Maintenance

Healthy PF carp juveniles (102 days old, 104.69 ± 3.08 g in weight, 14.65 ± 0.46 cm in length) were transported from Yugong Ecological Agricultural Technology Co., Ltd. (Qingtian, Lishui, Zhejiang, China) to the PF Carp Research Center (Qingtian, Lishui, Zhejiang, China), where they acclimated in circular tanks (104 cm × 80 cm, water volume: 1200 L) outfitted with electric heaters and water treatment equipment for 14 days. During the period of acclimation, fish were fed artificial food (1% of their body weight: 30% crude protein, 3% crude fat; Techbank, Ningbo, China) twice per day (7:00 a.m., 6:00 p.m.). Water temperature was controlled at 28 ± 0.5 °C and DO was maintained at 6.56 ± 0.20 mg/L.

### 2.2. Experimental Design and Sample Collection

After 14 days of acclimation, 200 robust and healthy fish were used for thermal stress trials. They were divided into one of three groups, each with three replicates. To roughly simulate the summertime temperature range in the rice fields, three temperatures (28 °C, 34 °C, and 38 °C) were chosen. Furthermore, in our preliminary experiment, we found that mass die-off of PF carp occurred at 39 °C, and they were in a state of stress at 38 °C. Consequently, the greatest threshold for the thermal stress temperature experiment was chosen to be 38 °C. Each treatment was replicated three times (20 fish per tank). The experiment lasted 24 h. The water conditions were kept constant, and the fish were fasted throughout the experiment.

For the control group, we randomly chose six fish from three tanks (three replicates for each tank) with a water temperature of 28 ± 0.5 °C at the beginning of the experiment (CG). The water conditions during the experiment were as follows: pH 7.8–8.2; 6 mg/L dissolved oxygen. After the test began, the water temperature was raised by 1 °C·h^−1^ until the goal temperatures for the experimental group (EG34 and EG38) were attained, and then the temperature was kept constant. Fish were collected 24 h after the goal temperature was obtained. At each goal temperature, six fish from each tank were randomly selected and were anesthetized with 0.3 mg/L MS-222 (Sigma, St. Louis, MO, USA). The liver tissues were collected right away. All samples were frozen right away with liquid nitrogen and kept at −80 °C.

### 2.3. Metabolite Extraction

For each group, six samples were chosen for the metabolomics test, and the samples were thawed in stages from −80 °C to −20 °C in an ice-water bath. Each sample was weighed to 50 mg in total, and then placed in EP tubes. Once the methanol/water (4:1, *v*/*v*) mixture was precooled, 400 µL was added to all EP tubes, and the tissue crushers were used to crush the samples (−20 °C, 50 Hz). The samples were allowed to settle for 30 min at 20 °C. Next, the supernatant was centrifuged (parameters: 4 °C, 13,000× *g*, and 15 min), and then stored in the LC–MS injection vial for further metabolomics analysis. LC–MS analysis of tissue samples was performed using methanol, acetonitrile (LC–MS grade), and formic acid (LC–MS grade), all ordered from Fisher Scientific (Hampton, NH, USA).

### 2.4. LC–MS Detection

The LC–MS analysis was performed using a Thermo Q Exactive Mass Spectrometer (Thermo Scientific, San Jose, CA, USA) with an electrospray interface. The UHPLC system had a binary solvent delivery manager and a sample manager. The chromatographic parameters were as follows: ethylene-bridged hybrid C18 column (100 mm × 2.1 mm, 1.7 μm, Waters, Milford, MA, USA); mobile phase gradient, (A) deionized water with 0.1% formic acid and (B) acetonitrile/isopropanol (1:1, *v*/*v*) mixture with 0.1% formic acid; flow rate, 0.4 mL/min; sample injection volume, 10 μL; and column temperature, 40 °C. The mobile phase gradient was as follows: 0–3 min, 95–80% A; 3–9 min, 80–5% A; 9–13 min, 5–5% A; 13–13.1 min, 5–95% A; and 13.1–16 min, 95–95% A. The MS conditions were as follows: scan range (*m*/*z*), 70–1050; flow rate of sheath gas, 40 psi; flow rate of auxiliary gas, 10 psi; temperature of auxiliary gas, 400 °C; normalized collision energy, 20–40–60 V; and IonSpray floating voltage, positive mode (ESI+), +3500 V and negative mode (ESI−), −2800 V. During the experiment, in addition to the six analytical samples, a QC sample was introduced to assess the stability of the analytical system and the reproducibility of the results.

### 2.5. Data Processing and Differential Metabolite Identification

The Progenesis QI software (Waters 515, Milford, MA, USA) was used to clean up the original data from all LC–MS analyses. For retrieval, the HMDB database (http://www.hmdb.ca/accessed on 1 October 2022), the METLIN database (https://metlin.scripps.edu/ accessed on 10 October 2022), the KEGG database (https://www.genome.jp/kegg/ accessed on 15 October 2022), and a custom database were chosen. ROPLS software (v1.6.2) was used to perform multivariate analyses such as principal component analysis (PCA) and orthogonal partial least-squares discrimination analysis (OPLS-DA) (v1.6.2). In addition, the OPLS-DA models were validated using a 200-permutation test. The OPLS-DA model’s VIP values of projected metabolites (VIP, VIP > 1) and the *p*-value tests (*p* < 0.05) were used as the screening criteria to select differential metabolites and possible biomarkers. The modified metabolites were analyzed for their place in the KEGG pathway using Metabo Analyst 4.0 [43] from the metabolomic profiling platform and Fisher’s exact test. Using the Benjamini and Hochberg (BH) method, the *p*-values were adjusted. The significance threshold for enrichment was determined as a *p*-value of <0.05 and an impact value of >0.1.

## 3. Results

### 3.1. Total Ion Map Analysis of Metabolites

**Figure 2:**
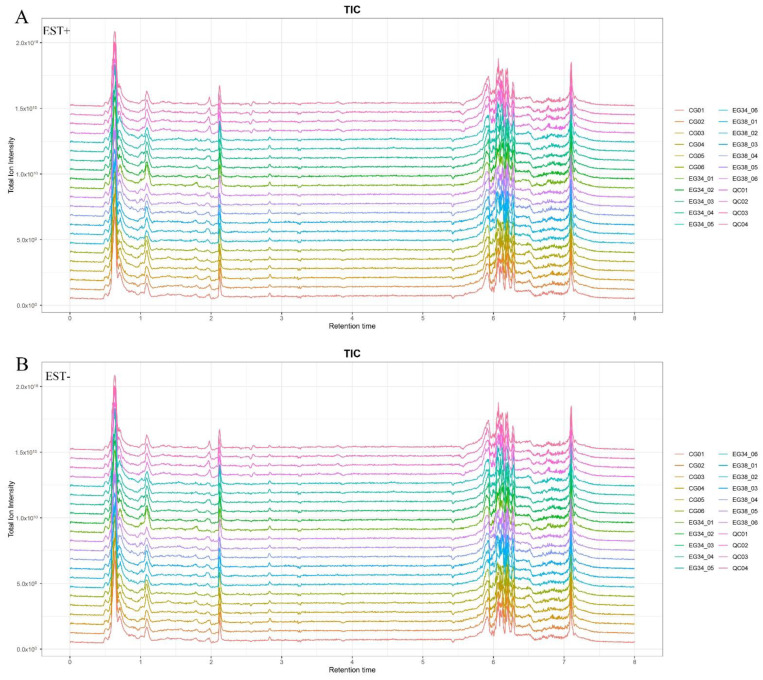
The total ion current (TIC) of liver samples from *Cyprinus carpio var qingtianensis* measured in (**A**) positive (ESI+) and (**B**) negative (ESI−) modes. CG01–CG06: six samples from control group; EG34_01–EG34_01: six samples from experimental group (34 °C) under thermal stress; EG38_01–EG38_06: six samples from experimental group (38 °C) under thermal stress; and QC01–QC04: four samples from quality control group.

The positive ion mode (ESI+) and negative ion mode (ESI−) UPLC-Q Exactive total ion current chromatograms are shown in Figure 2. Our LC–MS system’s high reproducibility can be seen from the overlapping QC sample total ion current (TIC) chromatograms. The chromatographic peak response intensity was also shown to have the same response strength and retention time. This confirms the reliability of the UHPLC-Q Exactive analysis platform detection results. In ESI+ ion mode, 4509 features were found; in ESI− ion mode, 4471 features were found.

### 3.2. Statistical Analysis of Data

**Figure 3:**
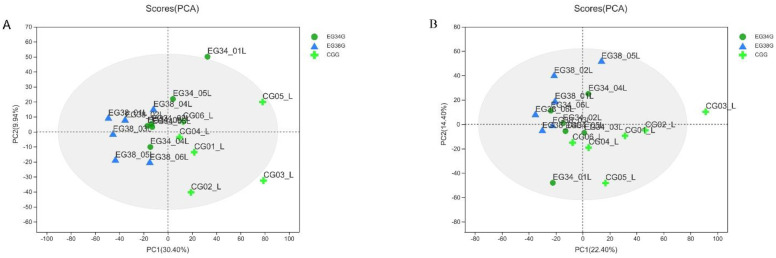
PCA score plot for samples from the treatment and QC groups: (**A**) ESI+; (**B**) ESI−.

**Figure 4:**
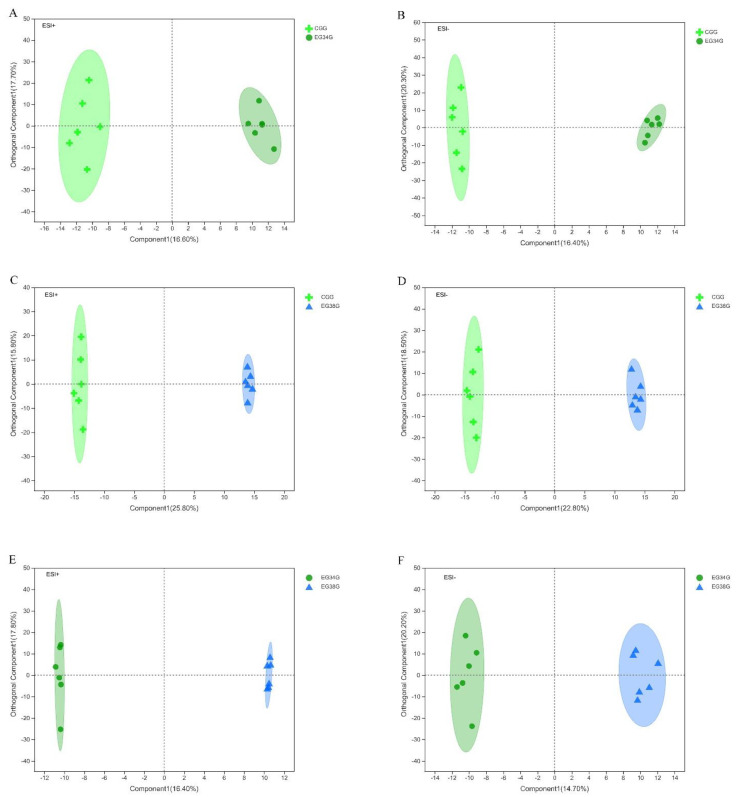
OPLS−DA score scatter plots: (**A**) CG vs. EG34 (ESI+); (**B**) CG vs. EG34 (ESI−); (**C**) CG vs. EG38 (ESI+); (**D**) CG vs. EG38 (ESI−); (**E**) EG34 vs. EG38 (ESI+); and (**F**) EG34 vs. EG38 (ESI−).

After the data were normalized, unsupervised multivariate analysis (PCA) and supervised analysis (OPLS-DA) were used to evaluate the quality control (QC) samples and other investigational samples. Principal component analysis (PCA) was performed on the data set and showed that all groups were within the 95% confidence interval (Hotelling’s T-squared ellipse). In positive and negative modes, the PCA model’s R^2^X predictive ability values were 0.434 and 0.402, respectively, indicating that the information was statistically trustworthy (Figure 3). Therefore, a PLS-DA model was used to further investigate how the groups differed. The parameter R^2^Y denotes the model’s interpretation rate, and Q^2^ denotes the model’s prediction rate. Typically, a parameter larger than 0.4 is necessary for a viable model. The results of the OPLS-DA score plots for the CG vs. EG34, CG vs. EG38, and EG34 vs. EG38 groups in positive and negative modes were as follows: ESI+: R^2^X = 0.927, R^2^Y = 0.949, R^2^Y = 0.942, Q^2^ = 0.542, Q^2^ = 0.842, Q^2^ = 0.591; ESI−: R^2^X = 0.947, R*^2^*Y = 0.947, R^2^Y = 0.938, Q^2^ = 0.569, Q^2^ = 0.828, and Q^2^ = 0.682. Therefore, the cumulative R^2^Y and Q^2^ values of the OPLS-DA model in ESI+ and ESI− modes were both above 0.50, highlighting the predictive performance and reliability of the models. Figure 4 shows that each comparison group’s sample scatter points could be clustered on both sides of the T score [1] axis. This indicates that the differences between the comparison groups were clear, and that the OPLS-DA model could effectively distinguish the metabolites between groups. Lastly, VIP values of >1 (OPLS-DA model) and *p*-values of <0.05 could be used to screen for different metabolites in the comparison groups (one-way ANOVA).

### 3.3. Screening of Differential Metabolites

**Figure 5:**
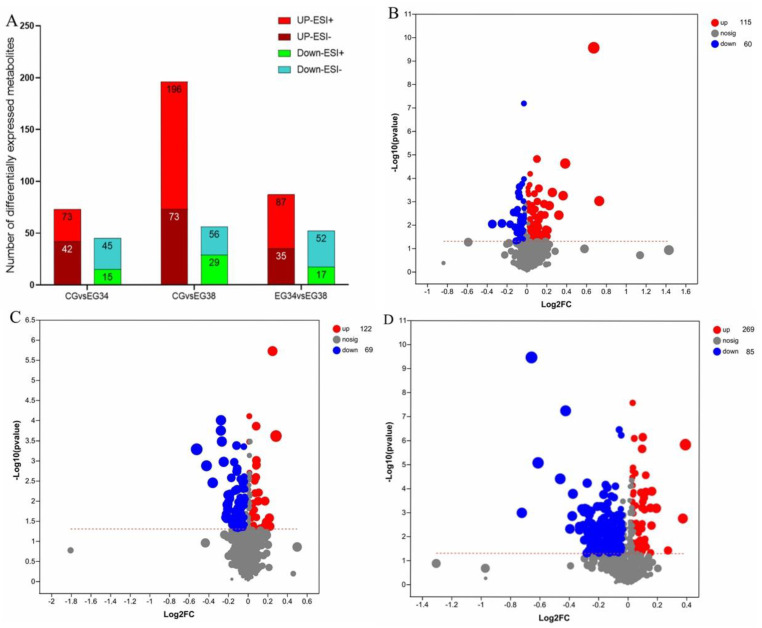
Differentially expressed metabolites in three comparison groups: (**A**) summary of the various metabolites in the two comparison groups; (**B**) volcano plot of the differentially expressed metabolites in the CG and EG34 groups; (**C**) volcano plot of the differentially expressed metabolites in the CG and EG38 groups; and (**D**) volcano plot of the differentially displayed metabolites in the EG34 and EG38 groups.

The VIP in the OPLS-DA model (VIP > 1) and the *p*-value of Student’s *t*-test (*p* < 0.05) were used to determine the different metabolites. To identify various metabolites, a Human Metabolome Database (HMDB, http://www.hmdb.ca/ accessed on 10 October 2022) information search was employed. A total of 115 upregulated and 60 downregulated metabolites were screened and identified in the CG vs. EG34 group, yielding a total of 175 differentially expressed metabolites (DEMs). The detected metabolites mainly included guanosine, xanthine, ornithine, homodolichosterone, d-ornithine, phosphatidylcholine (PC (22:4 (7Z,10Z,13Z,16Z)/P-16:0)); PC (16:0/20:4 (5Z,8Z,11Z,14Z)); PC (22:6 (4Z,7Z,10Z,13Z,16Z,19Z)/16:0); LysoPC (22:4 (7Z,10Z,13Z,16Z)/0:0); LysoPC (22:5 (4Z,7Z,10Z,13Z,16Z)/0:0)), l-2-aminoadipic acid, and caffeic acid. A total of 354 DEMs were screened in the CG vs. EG38 groups, of which 85 metabolites were downregulated and 269 metabolites were upregulated. The metabolites mainly included ornithine, guanosine, docosahexaenoic acid, l-glutamine, prostaglandin F1a, N−methyl−L−glutamic acid, phosphatidylcholine (PC (16:1 (9Z)/20:4 (5Z,8Z,11Z,14Z)), PC (16:0/20:4 (5Z,8Z,11Z,14Z)), PC (22:6 (4Z,7Z,10Z,13Z,16Z,19Z)/16:0), LysoPC (22:4 (7Z,10Z,13Z,16Z)/0:0), LysoPC (20:0/0:0), LysoPC (22:5 (4Z,7Z,10Z,13Z,16Z)/0:0), PC (22:6 (4Z,7Z,10Z,13Z,16Z,19Z)/16:0)), PE (18:1 (11Z)/18:1 (9Z)), Sph, valine, spermidine, aminoacetone, 3-hydroxy-*N*^6^,*N*^6^,*N*^6^-trimethyl-l-lysine (TML), hexanoic acid, d-glutamine, and ethyl beta-d-glucopyranoside. The main DEMs identified in the EG34 vs. EG38 groups were 3-hydroxylidocaine, LysoPC (22:5 (4Z,7Z,10Z,13Z,16Z)/0:0), 3-oxoglutaric acid, phenylalanylglutamic acid, glycerylphosphorylethanolamine (GPC), spermidine, ornithine, pyroglutamic acid, and *N*-acetylneuraminic acid (Figure 5). Of these, most metabolism-related genes were involved in lipid metabolism, amino-acid metabolism, and carbohydrate metabolism (Table 1).

### 3.4. Differential Metabolite KEGG Pathway Analysis

**Figure 6:**
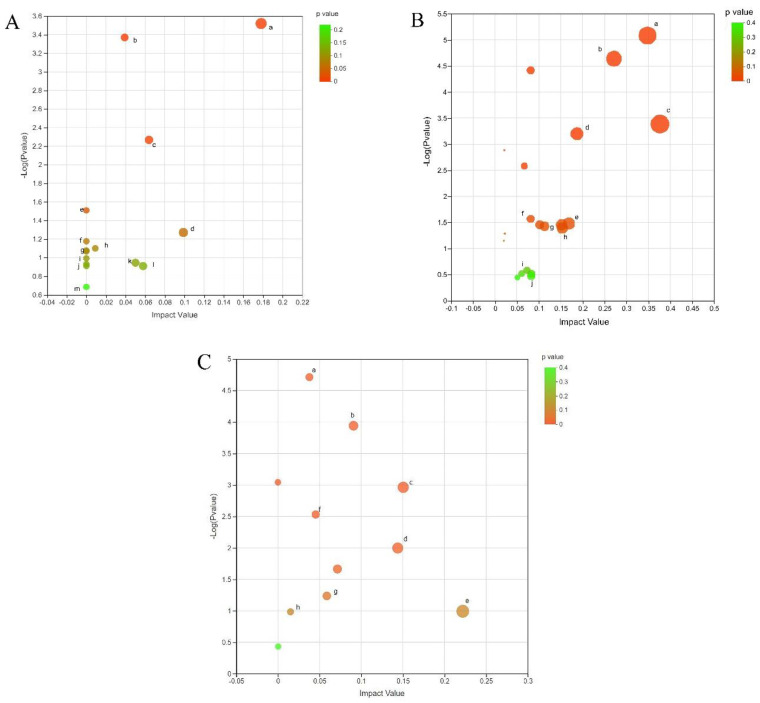
(**A**) DEM metabolic pathway enrichment in CG vs. EG34 groups (KEGG Topology Analysis). Note: a, arginine and proline metabolism; b, purine metabolism; c, glycerophospholipid metabolism; d, arginine biosynthesis; e, linoleic acid metabolism; f, alpha–linolenic acid metabolism; g, glutathione metabolism; h, starch and sucrose metabolism; i, galactose metabolism; j, fatty acid biosynthesis; k, fructose and mannose metabolism; l, cysteine and methionine metabolism; and m, amino sugar and nucleotide sugar metabolism. (**B**) DEM metabolic pathway enrichment in CG vs. EG38 groups (KEGG Topology Analysis). Note: a, arginine biosynthesis; b, arginine and proline metabolism; c, alanine, aspartate, and glutamate metabolism; d, tryptophan metabolism; e, cysteine and methionine metabolism; f, glycerophospholipid metabolism; g, beta–alanine metabolism; h, lysine biosynthesis; and i, fructose and mannose metabolism. (**C**) DEM metabolic pathway enrichment in EG34 vs. EG38 groups (KEGG Topology Analysis). Note: a, amino sugar and nucleotide sugar metabolism; b, glycerophospholipid metabolism; c, arginine biosynthesis; d, arginine and proline metabolism; e, galactose metabolism; f, glutathione metabolism; g, beta–alanine metabolism; and h, glycine, serine, and threonine metabolism.

Using the MetaboAnalyst 4.0 metabolomics data comprehensive analysis platform to perform an enrichment analysis of the KEGG metabolic pathway for the screened differential metabolites, the results displayed that the 175 differential metabolites in the CG vs. E34 groups involved 26 metabolic pathways including arginine and proline metabolism, purine metabolism, glycerophospholipid metabolism, arginine biosynthesis, alpha-linolenic acid metabolism, linoleic acid metabolism, glutathione metabolism, starch and sucrose metabolism, galactose metabolism, fatty acid biosynthesis, cysteine and methionine metabolism, fructose and mannose metabolism, and amino sugar and nucleotide sugar metabolism (Figure 6A). The 354 differential metabolites in the CG vs. EG38 group were involved in 20 metabolic pathways, including arginine and proline metabolism, arginine biosynthesis, alanine, aspartate, and glutamate metabolism, tryptophan metabolism, cysteine and methionine metabolism, glycerophospholipid metabolism, beta-alanine metabolism, lysine biosynthesis, and fructose and mannose metabolism (Figure 6B). In the EG 34 vs. EG 38 group, the 191 differential metabolites involved 20 metabolic pathways, including amino sugar and nucleotide sugar metabolism, glycerophospholipid metabolism, arginine biosynthesis, arginine and proline metabolism, galactose metabolism, glutathione metabolism, beta-alanine metabolism, and glycine, serine, and threonine metabolism (Figure 6C).

## 4. Discussion

Water temperature has direct effects on the oxidative stress, blood parameters, and energy homeostasis of fish [44,45]. The liver, which plays an important role in metabolism and detoxification, often stops working when under thermal stress [46]. Excessive temperature leads to protein degeneration, protein degradation, and the heat-shock response in fish, with a number of physiological and metabolic effects on the central nervous system, immune system, digestive system, metabolic capacity, and antioxidant stress [47,48,49,50,51]. Previous research has shown that, a few days before a change in temperature, the liver of common carp (*Cyprinus carpio* L.) undergoes a switch to lipid metabolism [35]. Therefore, it is crucial to explore the liver metabolome under thermal stress. As a result, in the current study, LC–MS was used to screen metabolites in the liver of PF carp subjected to thermal stress, followed by KEGG pathway enrichment analyses. Most genes were involved in metabolism, including lipid, amino-acid, and carbohydrate metabolism. Similar metabolic pathways, including the metabolism of GPs, glutamine and glutamate, and purines, were activated in all three groups. To evaluate if the same metabolic pathway contributes to the same physiological metabolic function in different groups, we examined the direction of pathway regulation through metabolic pathways and various metabolites at key nodes. Several of the most important metabolites and the KEGG pathways to which they are linked are discussed below.

### 4.1. Lipid Metabolism

Our results show that heat stress regulates lipid metabolism and keeps metabolic homeostasis in check by altering gene expression and metabolite levels in juvenile PF carp exposed to varying thermal regimes. In the past, lipids have been thought of as one of the primary sources of energy that are found in the body [52]. However, in more recent times, it has been found that lipids also play an important role in the composition of cellular structure [53], the regulation of signaling factors [53], and the regulation of host immunity and metabolism [54,55]. Lipids are an important nutrient for microorganisms, fish, and other animals. There are eight types of lipids: fatty acyls, glycerolipids, glycerophospholipids, sphingolipids, prenol lipids, sterol lipids, saccharolipids, and polyketides [54,55]. The cell membrane supports the majority of enzymes and metabolism of intracellular materials [56,57,58]. Glycerophospholipids (GPs), a membrane component, are indispensable for metabolism and signal transduction [59]; the metabolites of the glycerophospholipid pathway such as phosphatidylcholine (PC, lecithin), phosphatidylethanolamine (PE), and phosphatidylserine are common GPs (PS). Different metabolite enrichment metabolic pathways showed that the content of seven types of PC (PC (16:0/22:6 (4Z,7Z,10Z,13Z,16Z,19Z)), PC (18:3 (9Z,12Z,15Z)/20:4 (8Z,11Z,14Z,17Z)), PC (22:6 (4Z,7Z,10Z,13Z,16Z,19Z)/16:0), PC (16:1 (9Z)/20:4 (5Z,8Z,11Z,14Z)), PC (18:3 (9Z,12Z,15Z)/20:4 (8Z,11Z,14Z,17Z)), PC (16:0/20:4 (5Z,8Z,11Z,14Z)), and PC (22:6 (4Z,7Z,10Z,13Z,16Z,19Z)/16:0)) decreased significantly after EG38. Accordingly, membrane fluidity decreased under high-temperature stress. PF carp maintained lipid metabolism by regulating genes and metabolites related to lipid metabolism, preserving membrane homeostasis, and suppressing lipid deposition. Previous research has shown that common carp (*Cyprinus carpio* L.) may regulate the composition of their cell membranes by downregulating genes involved in phospholipid metabolism [35]. According to the effects of high temperature on the metabolism of juvenile turbot (*Scophthalmus maximus*), it was also found that high temperature affected the expression of genes related to lipid metabolism, affected the structure of the cell membrane, and led to the reduction in lipid compounds, for example, glycerol and phospholipid, which may be an effective strategy for alleviating fat deposition caused by high temperature [10]. In addition, arachidonic acid (ARA), via the actions of phospholipase A2 (PLA2), linolenic acid (LA), and lysophosphatidyl cholines (LysoPCs) is important; the metabolism of GP exhibits a dynamic equilibrium between synthesis and deconstruction, with the formation of ARA being a major catabolic route for LA and LysoPCs under the action of PLA2 [60,61]. In this investigation, five LysoPCs (LysoPC (22:4 (7Z,10Z,13Z,16Z)/0:0), LysoPC (22:5 (4Z,7Z,10Z,13Z,16Z)/0:0), LysoPC (20:2(11Z,14Z)/0:0), LysoPC (22:4 (7Z,10Z,13Z,16Z)/0:0), and LysoPC (22:5 (4Z,7Z,10Z,13Z,16Z)/0:0)) were discovered. The metabolic pathways for ARA and LA in the EG38 group were triggered by thermal stress. Sphingolipid (SM) levels were also found to vary dramatically under acute heat stress. Phosphocholines and ceramide can be generated when SM is hydrolyzed by sphingomyelinase (SMase). Ceramidase (CDase) is responsible for the hydrolysis of ceramide, resulting in sphingosine 1-phosphate (Sph) and free fatty acids [62]. Inhibiting PkC activity via the TNF pathway, ceramide can function as a second messenger (tumor necrosis factor pathway) [63]. It disrupts the regular rhythmic processes that occur on a cellular level including differentiation, apoptosis, and proliferation [64]. The alcohol backbone of intracellular sphingolipids can be phosphorylated by sphingosine kinase to create sphingosine 1 phosphate (S1P). Sph is commonly considered as a negative regulatory marker of the apoptosis signaling pathway because of studies showing that the process via which Sph phosphorylation produces S1P can effectively inhibit cell apoptosis [65]. The results of this study revealed that there was a significant decrease in Sph content and an increase in SM content under thermal stress. This suggests that, under conditions of thermal stress, liver cells inhibit SMase activity, reduce the formation of ceramide, and promote cell apoptosis through enhanced PkC activity; concurrently, the considerable drop in Sph concentration confirms accelerated hepatopancreas cell apoptosis.

### 4.2. Amino-Acid Metabolism

High temperatures have been linked to elevated ammonia levels in fish, which are produced by the catabolism of proteins and amino acids in the liver [66]. Amino acids are among the key substrates for energy synthesis in fish, and they are obtained either from feed or via the breakdown of tissue proteins [67]. Amino acids, such as glutamate (Glu) and glutamine (Gln), are substrates for animals to synthesize protein and low-molecular-weight metabolites with enormous physiological importance. For fish, Glu and Gln serve crucial regulatory roles in metabolism, gene expression, and immunology [68]. As a primary energy source for leukocytes and a critical regulator of the generation of cytokines and NO, glutamine is vital to the immune response in fish [69]. Both glutamate and glutamine are important energy substrates in fish, but the tissue-specific metabolism of these two amino acids (AA) in aquatic organisms has not been fully characterized [68]. The amounts of Glu and Gln metabolites considerably increased in the EG34 and EG38 groups after 24 h of temperature stress in this experiment, suggesting that there may have been an accumulation of body ammonia due to amino-acid catabolism. This is consistent with earlier research [11]. An increased tissue content of ammonia is harmful to fish health since it is neurotoxic. Fish exposed to extreme temperatures must maintain a high concentration of ammonia in their bodies to survive [70]. Glu and Gln are nonessential amino acids (NEAA) found in fish. Glutamine is made from glutamate by the enzyme glutamine synthetase and converted back to glutamate by the enzyme glutaminase [71]. In particular, the prevalence of deamination, which leads to the production of ammonia as a result of amino-acid catabolism, distinguishes fish glutamate metabolism from the metabolic process in mammals [72]. In contrast to most other amino acids, the levels of Glu and Gln showed a significant increase in the current study. These results were different from those found in salmonid species in earlier studies (e.g., Atlantic salmon and steelhead trout) [73,74]. This discrepancy may be due to the higher water temperature in the current study (38 °C) compared with the previous studies (18 °C and 20 °C). Similarly, the study conducted by Liu et al. (2019) further supports this point [11]. As one of the indispensable amino acids for fish, arginine plays important roles in the regulation of metabolism and immunity in animals [75]. Arginine is one of the most versatile amino acids because it is employed as a precursor in the biosynthesis of many different compounds in terrestrial animals, such as protein, nitric oxide (NO), urea, polyamines, proline, creatine, glutamate, and agmatine [76]. The arginine content of PF carp decreased significantly after thermal stress (EG38), indicating that the reduction in amino acids may lead to a consequent decrease in the synthesis of some proteins. Another study conducted on the large yellow croaker (*Larimichthy crocea*) showed consistent results after heat stress [77]. Tetrahydrobiopterin-dependent NO synthase (NOS; (Ja. and Dm.)) in fish is responsible for NO production (NOS and arginase convert urea and ornithine) [78,79]. Ornithine can be used to generate proline or polyamines. The plasma levels of arginine and ornithine declined considerably under thermal stress in *Senegalese sole* [80]. Additionally, excess transamination causes the liver to create NH_4_, which has been demonstrated to be hazardous to fish [81]. The levels of ornithine and N-acetyl-L-glutamic acid were found to be significantly higher in the 24 h thermal stress group in this study. N-Acetyl-L-glutamic acid is a cofactor of carbamoyl phosphate synthase I, which helps it accelerate the production of glutamate [82]. Xarbamoyl phosphate is synthesized from NH_4_, CO_2_, and water, and it enters the route of ornithine and arginine metabolism. This cycle starts with arginine, which is broken down by arginase into ornithine and urea [83]. In addition, we discovered a large increase in ubiquitin levels in the pathway involved in the metabolism of amino acids. Madeira et al. also found that the amount of total ubiquitin in *Sparus aurata* increased at higher temperatures, which was a sign of protein damage [84]. Feidantsis et al. discovered an increase in ubiquitinated protein levels in the heart and liver of gilthead seabreams after combined warming and hypercapnia (*Sparus aurata*) [85]. Our findings indicate that high-temperature exposure promotes the increase in ubiquitin content, thus enabling protein interpretation.

### 4.3. Carbohydrate Metabolism

Many DEMs were found to be enriched for genes involved in carbohydrate biosynthesis, as determined by KEGG enrichment analysis. When the body requires a steady supply of energy, liver glycogen is quickly depleted [86], along with the sugar metabolism products downstream. Carbohydrates can be catabolized for energy (ATP) or used for anabolic functions such as fatty acid production [87]. Fructose is primarily metabolized by the liver. Glyceraldehyde is phosphorylated by triose kinase into the glycolytic intermediate glyceraldehyde 3-phosphate. The biosynthetic pathway, which includes the generation of fatty acids and glycogen storage, can be continued via glycolytic intermediates. At a certain temperature (Tc), the imbalance between oxygen production and consumption becomes too significant, and anaerobic metabolism starts [88]. D-Gluconic acid is present in the body in the physiologically relevant form of gluconate, which is produced by the gluconate shunt via the action of glucose dehydrogenase (also known as glucose oxidase). In our study, glucose and D-gluconic content was found to increase significantly under high-temperature stress (34 °C and 38 °C), indicating that an unfavorable environment forces PF carp to switch to gluconeogenesis or glycogenolysis, resulting in glucose utilization by somatic cells. Previous research revealed that the glycogen content of *Notothenia rossii* remained unchanged at 8 °C, whereas *Notothenia coriiceps* demonstrated a substantial increase in glycogen after 6 h of temperature stress [89]. This further demonstrates that the effect of heat stress on aquatic species is strongly correlated to exposure duration. In addition, we found that citric acid was involved in the TCA cycle in the EG38 group. The content of citric acid in the EG38 group was significantly higher than that in the CG. We assume that, in the EG38 group, citric acid was implicated in the TCA cycle, which provides the reduction equivalents for terminal oxidation. More TCA cycle activity indicates a higher need for energy and a faster metabolic rate. Song et al. found that, when black rockfish (*Sebastes schlegelii*) were exposed to heat stress (27 °C), the amount of citric acid in their bodies increased substantially, and the TCA cycle led to more ATP being generated to help the fish deal with stressful conditions [23]. Carbamyol phosphate synthase I uses *N*-acetyl-neuraminic acid as a cofactor, which in turn stimulates the enzyme to catalyze the reaction of glutamate, ammonium hydroxide (NH4), carbon monoxide (CO), and water, yielding carbamoyl phosphate and allowing the cell to proceed down the ornithine and arginine metabolism pathway [82]. When thermal stress is reintroduced, the levels of ornithine and N-acetyl-L-neuraminic acid in the liver of PF carp are dramatically decreased, confirming that thermal stress induced a weakening of transamination in the process.

## 5. Conclusions

Despite numerous findings on metabolic and molecular responses to temperature stress in other fish, our knowledge of PF carp responses to thermal stress is extremely limited. In this study, metabolomic profiling analysis demonstrated that thermal stress in PF carp has profound effects on biological processes in the liver, with many pathways involved in carbohydrate metabolism, lipid metabolism, and amino-acid metabolism. Furthermore, the metabolic level adaptation and regulation mechanism of PF carp during thermal stress changes were discussed. Further experimental trials are required to obtain a thorough description of the physiological control mechanism in PF carp during heat stress, and the ensuing analysis must be integrated with other omics studies in order to identify significant and unique metabolic pathways.

## Figures and Tables

**Table 1:** Significantly different metabolites.

Metabolic Pathway (KEGG Pathway)	Metabolite	ESI+/−	Rt (min)	*m*/*z*	VIP Value	*p*-Value	Up/Downregulated		
							CG vs. EG34	CG vs. EG38	EG34 vs. EG38
Carbohydrate metabolism	Stachyose	ESI+	1.011	684.25	1.07	0.017	↑	-	-
	d-Fructose	ESI+	0.636	213.09	2.36	0.010	-	↑	↑
	d-Ribulose 5-phosphate	ESI+	0.926	211.00	1.05	0.042	-	↑	-
Amino-acid metabolism	*N*^2^-Acetylornithine	ESI+	1.814	175.10	1.81	0.027	↑	↓	-
	Gluconic acid	ESI−	0.648	195.05	1.13	0.025	↑	↑	↑
	Glucose	ESI+	0.538	132.09	1.02	0.002	↑	↑	-
	3-Dehydroquinic acid	ESI+	1.954	191.05	2.70	0.010	-	↓	↓
	Glutamate (Glu) and glutamine	ESI−	5.70	506.02	2.09	0.05	↑	↑	-
	*N*-Acetyl-neuraminic acid	ESI−	0.68	160.06	3.10	0.035	-	↑	↓
	*O*-Phospho-l-serine	ESI−	0.608	184.00	1.17	0.042	-	↓	-
	Ornithine	ESI+	0.536	133.09	1.23	0.002		↑	
Lipid metabolism	PC (18:4/0:0)	ESI+	5.97	516.30	2.29	0.050	↓	-	-
	PC (15:0/0:0)	ESI+	6.106	504.30	1.127	0.007	↓	↑	-
	PC (18:3 (9Z,12Z,15Z)/20:4 (8Z,11Z,14Z,17Z))	ESI+	6.66	804.55	1.766	0.001	↑	↓	↓
	PC (16:0/22:6 (4Z,7Z,10Z,13Z,16Z,19Z))	ESI+	6.787	806.56	1.32	0.007	↓	-	-
	PC (16:0/20:4 (5Z,8Z,11Z,14Z))	ESI+	6.739	826.56	1.147	0.008	↓	↑	-
	PC (22:6 (4Z,7Z,10Z,13Z,16Z,19Z)/16:0)	ESI+	6.593	850.56	1.082	0.034	-	↓	-
	LysoPC (16:0/0:0)	ESI−	7.15	540.33	1.17	0.015	↓	-	-
	LysoPC (17:0/0:0)	ESI+	6.477	532.33	1.244	0.048	↑	↓	-
	LysoPC (20:2 (11Z,14Z)/0:0)	ESI+	6.302	570.35	1.205	0.005	-	↓	-
	LysoPC (22:4 (7Z,10Z,13Z,16Z)/0:0)	ESI+	6.184	572.37	1.79	0.002	↑	↑	-
	LysoPE (0:0/18:1 (11Z))	ESI+	6.293	524.30	1.708	0.020	-	↓	-
	LysoPE (0:0/20:1 (11Z))	ESI+	6.022	528.31	1.105	0.022	↑	↓	↑
	LysoPE (20:4 (5Z,8Z,11Z,14Z)/0:0)	ESI+	5.985	500.28	1.449	0.002	-	↓	-
	LysoPE (0:0/22:4 (7Z,10Z,13Z,16Z))	ESI+	5.933	562.34	1.866	0.006	-	↓	-
	LysoPE (22:6 (4Z,7Z,10Z,13Z,16Z,19Z)/0:0)	ESI−	5.963	524.80	1.072	0.016	-	↓	-
	LysoPI (20:4 (5Z,8Z,11Z,14Z)/0:0)	ESI−	6.476	619.29	2.265	0.002	-	↓	-
	LysoPI (18:1 (9Z)/0:0)	ESI+	7.144	619.29	2.034	0.001	-	↓	-
	Choline phosphate	ESI+	0.598	184.07	1.10	0.002	-	↓	-
	Dodecanoic acid	ESI+	5.24	218.21	2.11	0.032	↑	-	-
	Cortolone	ESI+	6.011	399.27	1.02	0.023	-	↑	-

PC: Phosphatidylcholine; LysoPC: Lyso phosphatidylcholine; LysoPE: Lyso phosphatidylethanolamine; LysoPI: Lyso phosphatidylinositol; “↑” indicates a significant increase, “↓” indicates a significant reduction, “-” indicates no significant difference.

## Data Availability

The datasets generated during and/or analyzed during the current study are available from the corresponding author on reasonable request.
